# The application of modular multifunctional left heart bypass circuit system integrated with ultrafiltration in thoracoabdominal aortic aneurysm repair

**DOI:** 10.3389/fcvm.2022.944287

**Published:** 2022-09-21

**Authors:** Lingjin Huang, Xuliang Chen, Qinghua Hu, Fanyan Luo, Jiajia Hu, Lian Duan, E. Wang, Zhi Ye, Chengliang Zhang

**Affiliations:** ^1^Department of Cardiovascular Surgery, Xiangya Hospital, Central South University, Changsha, China; ^2^National Clinical Research Center for Geriatric Disorders, Xiangya Hospital, Central South University, Changsha, China; ^3^Department of Anesthesiology, Xiangya Hospital, Central South University, Changsha, China

**Keywords:** thoracoabdominal aortic aneurysm repair, left heart bypass, cardiopulmonary bypass, hospital volume, spinal cord protection, selective visceral perfusion, kidney protection

## Abstract

Open thoracoabdominal aortic aneurysm (TAAA) repair is a complex and challenging operation with a high incidence of serious complications, and high perioperative mortality and morbidity. Left heart bypass (LHB) is a circulatory support system used to perfuse the distal aorta during TAAA operation, and the advantages of LHB include guaranteeing distal perfusion, reducing the use of heparin, and diminishing the risk of bleeding and postoperative neurological deficits. In China, the circuit for TAAA repair is deficient, and far from the perfusion requirements. We designed a modular multifunctional LHB circuit for TAAA repair. The modular circuit consisted of cannulation pipelines, functional consumables connection pipelines, and accessory pipelines. The accessory pipelines make up lines for selective visceral perfusion and kidney perfusion, suckers and rapid infusion. The circuit can be assembled according to surgical requirements. The ultrafilter and heat exchanger are integrated into the circuit to fulfill the basic demands of LHB. The LHB circuit also has pipelines for selective visceral perfusion to the celiac artery and superior mesenteric artery and renal perfusion pipelines. Meanwhile, the reserved pipelines facilitate the quick switch from LHB to conventional cardiopulmonary bypass (CPB). The reserved pipelines reduce the time of reassembling the CPB circuit. Moreover, the rapid infusion was integrated into the LHB circuit, which can rapid infusion when massive hemorrhage during the open procedures such as exposure and reconstruction of the aorta. The ultrafiltration can diminish the consequent hemodilution of hemorrhage and rapid infusion. A hemoperfusion cartridge also can be added to reduce the systemic inflammatory during operation. The circuit can meet the needs of LHB and quickly switch to conventional CPB. No oxygenator was required during LHB, which reduce the use of heparin and reduce the risk of bleeding. The heat exchanger contributes to temperature regulation; ultrafiltration, arterial filter, and rapid-infusion facilitated the blood volume management and are useful to maintain hemodynamic stability. This circuit made the assembly of the LHB circuit more easily, and more efficient, which may contribute to the TAAA repair operation performed in lower volume centers easily. 26 patients who received TAAA repair under the modular multifunctional LHB from January 2018-March 2022 were analyzed, and we achieved acceptable clinical outcomes. The in-hospital mortality and 30-day postoperative mortality were 15.4%, and the postoperative incidences of paraparesis (4%), stroke (4%), and AKI need hemodialysis (12%) were not particularly high, based on the limited patients sample size in short research period duration.

## Introduction

Open thoracoabdominal aortic aneurysm (TAAA) repair is a complex and challenging operation including extensive exposure and reconstruction of the thoracoabdominal aorta and vital aortic branches (1). Open TAAA repair has life-altering complications, and high perioperative mortality (2, 3), and the reported in-hospital mortality after TAAA repair was 15–25% (4, 5). It has been associated with an increased risk of late death and serious complications at specialized aortic centers (6, 7). The clinical outcomes were especially poor for patients undergoing urgent or emergent procedures at low-volume centers (4), and open TAAA repair remains a monumental undertaking limited to experienced surgeons and high-volume centers (6).

Left heart bypass (LHB) and cardiopulmonary bypass (CPB) with or without hypothermic circulatory arrest are important perfusion-assisted techniques strategy for organ protection in TAAA repair. LHB is a widespread circulatory support system used to perfuse the distal aorta during TAAA operation, and the advantages of LHB include guaranteeing distal perfusion, reducing the use of heparin and diminishing the risk of bleeding and postoperative neurological deficits (8, 9). The use of LHB was limited while the open TAAA repair developed rapidly in China, mainly due to the shortage of equipment and consumables, and the hospital volume. The circuit for TAAA repair is deficient, and far from the perfusion requirements.

We designed a modular multifunctional LHB circuit that can be assembled according to surgical requirements for TAAA repair.

### Indication

The LHB circuitry is modified to accommodate surgical needs specific to the surgical procedure. The LHB circuit is used for patients undergoing Crawford extent II TAAA repair to extend from the left subclavian artery down to the aortic bifurcation organ perfusion. LHB can provide isothermic self-oxygenated blood while the proximal anastomosis is being completed, so the cardiac function and pulmonary function should meet systemic oxygenation and perfusion.

### Selection of equipment

**FIGURE 1:**
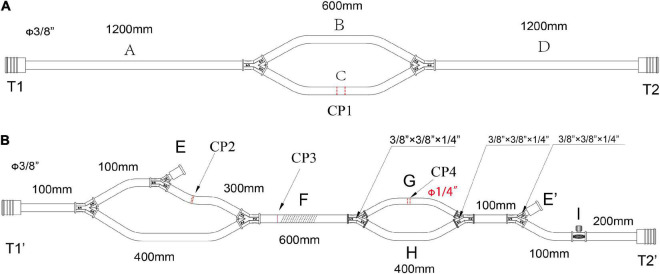
The main pipelines for cannulation and functional consumables connection in the LHB circuit. **(A)** The pipelines for cannulation, all diameters of the circuits are 3/8 inch, CP1 is the cut-point for connection of the cannulation of the inferior left pulmonary vein and the aortic cannulation. **(B)** The functional consumables connection pipelines of the LHB circuit. CP2 is the cut-point for the connection of a hard-shell reservoir, CP3 is the cut-point for a centrifugal pump, and CP4 for the heat exchanger. Line F can be occluded by a roller pump rather than a centrifugal pump in CP3. The port E and port E’ are a pair for an ultrafilter. Port I with a side hole is connected with a T-type adapter, which can be connected to a pressure monitor, and an arterial sampling connector. Both the port T1 in panel **(A)** and the port T1’ in panel **(B)** are connected with an oxygen saturation monitor or a connector. The port T2 in panel **(A)** and the port T2’ in panel **(B)** were connected with a Y-type connector (φ3/8” x3/8” x1/4”), and the 1/4-inch port of the connector is linked to the line for the selective visceral perfusion.

**FIGURE 2:**
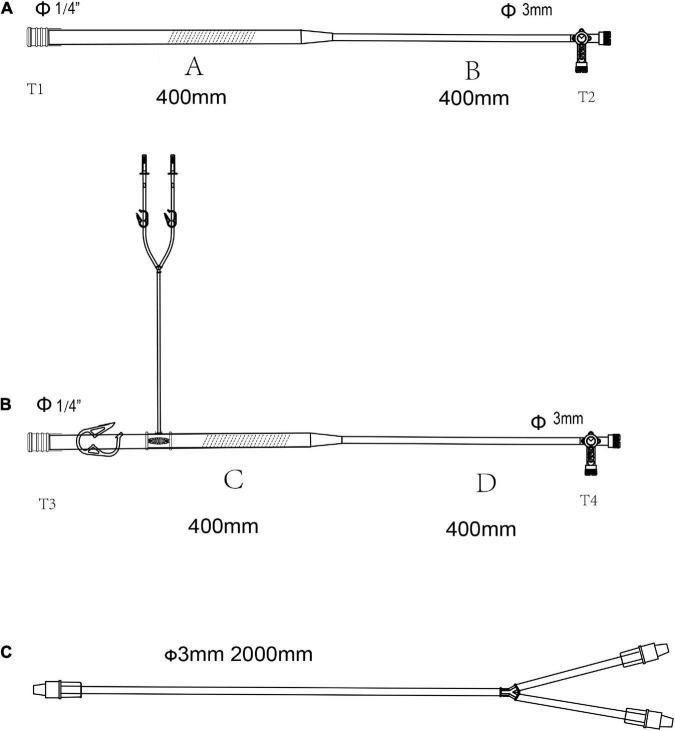
The pipelines for selective organ perfusion in the LHB circuit. **(A)** Line for selective visceral perfusion. **(B)** Line for selective kidney perfusion. **(C)** Y-type line connected to the T2 celiac axis for SMA and celiac axis perfusion or T4 to renal arteries for the selective perfusion. The line marked oblique dotted line is used for roller pumps.

**FIGURE 3:**
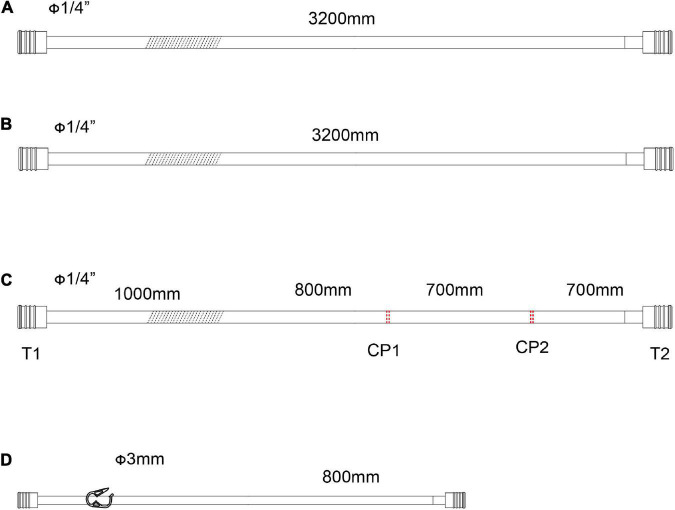
The accessory pipelines for shed blood collection, rapid infusion and blood sampling. **(A,B)** Pipeline for the sucker. The line marked oblique dotted line is used for roller pumps. **(C)** Accessory pipelines for rapid infusion, S1 is the cut-point for connection of ultrafilter, and S2 is the cut-point for an arterial filter. The line marked oblique dotted line is used for roller pumps. **(D)** The accessory pipelines for blood sampling.

**FIGURE 4:**
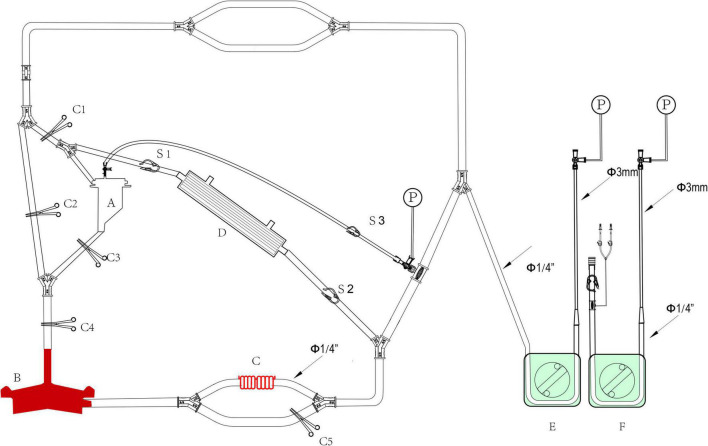
The assembled LHB circuit for perfusion. **A**, Hard-shell reservoir for priming and CPB reservation. **B**, centrifugal pump; **C**, heat exchanger; **D**, ultrafilter, **E,F**, roller pumps. **C1-C5**, Tube clumps; **S1-S3**, stop-flow clips.

**FIGURE 5:**
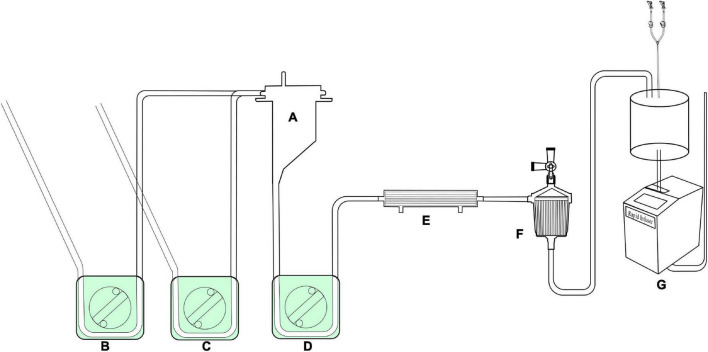
The assembled reserved LHB circuit for the sucker and rapid-infusion perfusion. **A**, Hard-shell reservoir for shed filed blood collection by the pump suckers. **B,C**, roller pumps for suckers; **D**, roller pump for shifting blood to the rapid-infusion system; **E**, ultrafilter, **F**, arterial filter, **G**, rapid-infusion system.

The circuit consists of reusable equipment and disposable. The pipelines of the LHB circuit mainly consist of three supplied sterile and individually wrapped bags as shown in Figures 1–5. Other disposable equipment includes a heat exchanger, two hard-shell reservoirs, a centrifugal pump, an ultrafilter, and arterial filter, a hemoperfusion cartridge, an oxygen saturation monitor, suction tips, and three pressure monitors, connectors, and other subsidiary tubes. Reusable equipment such as a CPB console, cooler-heater, and rapid-infusion system.

### The structure of the left heart bypass circuit

The LHB circuit mainly includes three parts: cannulation pipelines, functional consumables connection pipelines, and accessory pipelines. The accessory pipelines make up lines for selective visceral perfusion, kidney perfusion, suckers and rapid infusion.

The cannulation pipelines are shown in Figure 1A. The diameter is 3/8 inch, and the length is marked as the captions. Parallel lines B and C are the cannulation sites and the reserved cannulation sites. Line C can be separated into two parts in the cut-point CP1, one port is connected to the cannulation of the inferior left pulmonary vein to establish a drainage line, and another port is connected to the aortic cannulation as a perfusion line in the distal descending thoracic aorta or the proximal abdominal aorta. Line B is reserved for CPB. When LHB was performed, line B is clamped without blood flow. When LHB needs to switch to CPB, line C is separated into two ports, one connects to venous cannulation or cavoatrial cannulation as venous drainage, and the other port connects to another arterial cannulation as a perfusion line. The ports T1 and T2 in the terminal can be connected with the corresponded ports T1’ and T2’ in Figure 2.

Figure 1B displays the functional consumables connection pipelines of the LHB circuit. The diameter is 3/8 inch, except for the line marked with the red caption that the diameter is 1/4, and the length is also marked as the captions. The site of CP2 separates into two ports, one port is connected to the inlet port of the hard-shell reservoir for the solution or blood drainage, and the other port is connected to the outlet port of the hard-shell reservoir, which is a source of fluid for rapid transfusion. The site of CP4 can be separated into two ports to connect to the heat exchanger. The line can be cut into two ports in site CP3 for the connection of a centrifugal pump. Meanwhile, line F also can be occluded by a roller pump rather than a centrifugal pump in CP3 as the force of perfusion when a centrifugal pump is hard to get. Connector I with a side hole is connected with a T-type **adapter, which can be connected to a** pressure monitor, and an arterial sampling connector. The sealed port E and port E’ were a pair of ports for an ultrafilter. Both the port T1 in Figure 1A and the port T1’ in Figure 1B are connected with an oxygen saturation monitor or a connector. The port T2 in Figure 1A and the port T2’ in Figure 1B were connected with a Y-type connector (φ3/8” *3/8” *1/4”), and the 1/4-inch port of the connector is linked to the T1 side of the line (Figure 2A) for the selective visceral perfusion to the celiac artery and superior mesenteric artery.

The lines are shown in Figures 2A,B are used for the selective visceral perfusion. The port T1 was connected to the 1/4-inch port of the T-type adapter connected to T2 in Figure 1A and T2’ in Figure 1B, and the T-type adapter on the side T2 is connected to the port of T1 in the Y-type line (Figure 2C) for the selective visceral perfusion, and also connected to a pressure monitor. The tubes in Figure 3 with 1/4-inch are occluded by roller pumps, **t**he T-type adapter on the right side is connected to a pressure monitor, and also connect to the port T1 (Figure 2C) of the Y-type line for kidney perfusion.

As shown in Figures 3A,B, two lines for the sucker are connected to sucker tips to collect the shed blood to another hard-shell reservoir, which is used for autologous blood transfusion and rapid infusion. Figure 3C displays the reserved pipelines for rapid fusion, which are connected with an arterial filter and an ultrafilter, and collected the blood to the rapid-fusion system by a roller pump. The joint points S1 and S2 are connected with ultrafiltration and arterial filter, respectively. The terminal site marked T2 is connected to the rapid-infusion system. Figure 3D is used for blood sampling, which is the link between site I in Figure 1B and the inlet port of the hard-shell reservoir for perfusion.

### Assemble the circuit

The whole circuit can be assembled as previously described above. Briefly, the ports T1 and T1’ are a pair that can be connected by an adapter, T2 and T2’ in Figure 1 are connected by a Y-type three-way connector as well. A hard-shell reservoir inset to CP2 and a centrifugal pump add to CP3 when a centrifugal pump is used in the bypass. Line F marked with oblique dotted lines is occluded by a roller pump when the centrifugal pump cannot get. The heat exchanger is connected to CP4. Port I is connected by a pressure monitor and the sampling line in Figure 3D. The sealed port E and port E’ in Figure 1 are connected by an ultrafilter with a 1/4 inch tube for inflow and outflow. Each of the ports of T2 and T4 in Figure 2 is connected to one of the perfusion lines in Figure 2C. Each of the sucker lines is linked with a sucker tip and another hard-shell reservoir for autologous blood transfusion. T1 port in the rapid-fusion line (Figure 3C) connects to the outlet port of the hard-shell reservoir for autologous blood transfusion, while T2 connects to the rapid-fusion system. Meanwhile, an ultrafilter and an arterial filter are inserted into the sites of CP1 and CP2. The lines marked by the oblique dotted lines in Figure 3 are occluded by roller pumps for sucker or perfusion. The assembled circuit is shown in Figures 4, 5. The flow diagram was shown in Supplementary Video 1.

### Priming the circuit

**FIGURE 6:**
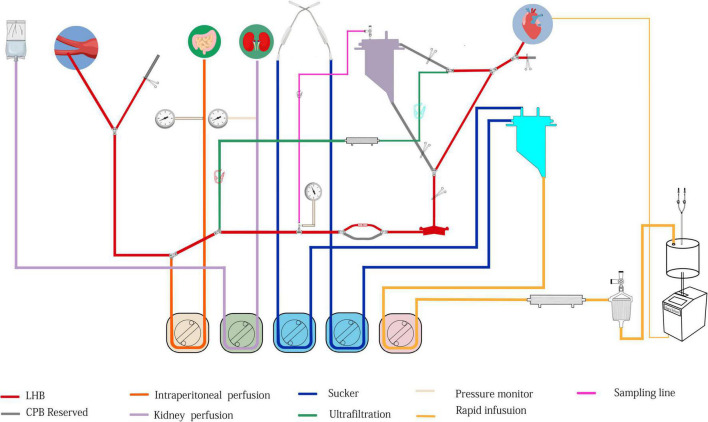
The structure of modular multifunctional LHB circuit integrated with ultrafiltration and reserved pipelines for TAAA repair. The functions were indicted by different colors at the bottom of the figure.

1,500 ml multiple electrolytes injection (plasma-lyte A) with 30 mg heparin was added to the hard-shell reservoir (A in Figure 4) as a priming solution. Once the connected devices are mounted on the CPB console, the water source to the heat exchanger (C in Figure 6) should be turned on. Start the pump to empty the circuit, loosen all the tube clumps (C1-C5 in Figure 4) and stop-flow clips (C1-C3 in Figure 4). The C2 is clamped when the line is full with solution, then start the centrifugal pump. Empty all the tubes and adjust the occlusion of the tube by the roller pumps for selective visceral perfusion. The microemboli gas in the ultrafilter, heater exchanger, and hemoperfusion cartridge should be eliminated. Warming the prime solution in the circuit by a water tank is an effective means to minimize the risk of releasing microbubbles of gas from the solution. Calibrate the zero values for pressure monitors and test the accuracy of the perfusion flow. Clamp all the tube clamps and stop-flow clips before bypassing initiating.

### Anesthesia management

All patients are intubated with a double-lumen endobronchial tube for later single right-lung ventilation. The blood pressures of the right radial or brachial for the upper limb monitoring, and the dorsal pedis artery for the lower limb in the opposite of the fusion cannulation in the iliac artery or femoral artery. A large-bore triple lumen central venous line is placed in the right internal jugular vein for central venous pressure monitoring, fluid infusion, anesthetics and vasoactive medications infusion, followed by the insertion of an 8.5F catheter sheath for fluid and blood rapid infusion. Transesophageal echocardiography (TEE) is also monitored to assess the hemodynamics and cardiac function changes. Cerebral oximetry is measured with near-infrared spectroscopy (NIRS), and the depth of anesthesia is also evaluated using the bispectral index (BIS). A temperature probe is positioned in the patient’s nasopharynx for temperature monitoring. Moreover, a urethral catheter integrated with a temperature probe is placed for urine output and temperature management. Self-adhesive defibrillation electrodes were located on the chest wall, and an external defibrillator is a standby. Cell-saving and rapid-infusion devices are set up so that shed blood can be salvaged and volume can be rapidly replaced, respectively. The baseline of activated *coagulation* time (ACT) and mixed venous oxygen saturation (SvO2) are examined when the central venous line is placed successfully.

### Left heart bypass management

#### Cannulation

The inferior left pulmonary vein is the routine site of atrial cannulation for the outflow line, and the proximal abdominal aorta, the iliac artery, or the femoral artery is selected to establish an inflow line. Heparin is administered intravenously at a dose of 1.5 mg/kg cannulation performs until the patient’s ACT is verified to be more than 280 s. A 22- or 24-Fr angled-tip venous cannula is connected to the outflow line of the LHB circuit, which is named C1 in Figure 1. Meanwhile, a 20- or 22-Fr straight tip arterial cannula is connected to port C2 in Figure 1 as a return line for LHB. Meanwhile, a Y-connector is attached to the return line that splits pump return between the line going to the distal aortic cannula and another line leading to the perfusion catheters for later delivery of selective visceral perfusion to the celiac artery and superior mesenteric artery (SMA).

### Conduct of left heart bypass

The LHB is initiated at a flow rate of 0.5 L/min after the ACT > 280 s, and the cannulation is performed and connected to the circuit correctly. Pay more attention to the drainage and perfusion pressure, the drainage is evaluated by the movement of cannulation in the pulmonary vein caused by the vacuum, and the perfusion pressure is read from the pressure monitor. The cannulation of pulmonary vein and artery are adjusted to maintain sufficient blood can be drained from the left heart to perfuse the lower body. The distal aortic perfusion mainly relays on the LHB flow rate when an aortic cross-clamp is applied. The flow rate is 2.5–3.5 L/min to keep the patient’s mean arterial pressure (MAP), both the upper limb and lower limb, maintain in the range of 70–90 mm Hg. The reserved pipeline marked B in Figure 2, which is paralleled with the line connected to the heat exchanger, can release to enhance the flow rate. Two suckers are used to collect the shed blood to the hard-shell reservoir, and then remove it to the rapid infuser system after ultrafiltration and filtering, which can rapidly infuse to main the volume. Meanwhile, volume and pharmacologic agents are adjusted to maintain an adequate MAP. Nasopharyngeal temperature and bladder temperature are monitored and adjust the water temperature in the heat exchangers regulates the body temperature according to the surgical processes. Ultrafiltration can be applied (Release the stop-flow clips C1 and C1 in Figure 6) when the volume is overloaded and excessive hemodilution.

Overall, the rapid infusion system the 8.5F catheter sheath can afford high to 1,000 ml/min warming fluid delivery to fulfillment of the volume demands of patients. Whereas the reserved rapid infusion modular will be applied, when the fusion flow is hard to successfully maintain the target MAP due to massive blood volume lost in the condition of catastrophic bleeding or aneurysm rupture. Our reserved hard-shell reservoir and pipelines can afford a more rapid infusion rate. Like the structure in Figure 6, partially or completely clamp the tube clump C2, and releasee the tube clump C3, which allows the blood or solution in the hard-shell reservoir (A in Figure 6) can be rapidly infused into the closed-loop pipeline of LHB circulation to enhance the blood volume. Blood gas and electrolyte analysis should be performed to evaluate the homeostasis during the LHB. The ACT should keep in a range of 250–400 s. 8% sodium citrate anticoagulant is used for the anticoagulation of the rapid fusion system, so serum sodium and serum calcium should be adjusted to the normal physiological ranges.

### Spinal cord protection

The intercostal and/or lumbar arteries, which are the main feeding artery of the spinal cord, should be revascularized with one branch of the aortic graft. During the reimplantation period, the MAP is kept relatively high (usually with a target MAP of 80–100 mm Hg) as part of a spinal protection strategy. Meanwhile, the temperature sustains at a relatively lower level near 34°C, which is another spinal cord protection strategy. It is better to enhance the hematocrit by ultrafiltration, and blood transfusion. Rewarming the patient to keep the Nasopharyngeal temperature near 36°C when the intercostal and/or lumbar arteries anastomosis is completed.

### Selective visceral perfusion and renal perfusion

Selective visceral perfusion and kidney perfusion are essential principles for TAAA repair during the abdominal aorta reconstruction, which aims to minimize the duration of abdominal-organ ischemia and reduce the incidence of renal and hepatic insufficiency, coagulopathy, and bowel ischemia. Selective visceral artery perfusion is performed with the oxygenated isothermic blood continuously delivered from the LHB circuit through a Y-type three-way connector of the perfusion line (E in Figure 6). A Y-branch off the line for selective is connected to the 10-Fr balloon catheters inserted into the celiac axis and the SMA. The oxygenated isothermic blood perfuses the celiac axis and the SMA at a flow rate of 500 mL/min, which is delivered continuously by a roller pump. Moreover, it is important to maintain the pressure of visceral perfusion to less than 200 mmHg. The flow halves when perfusion is applied only to the celiac axis or the SMA.

A renal perfusion system is used to maintain selective renal hypothermia for the preservation of renal function. The renal perfusion is conducted with the renal perfusion system, which is set up with a separated tube connected with a solution inlet, a Y-branch off the line connected with two10-Fr balloon catheters, which is placed in the renal arteries to accept the cold crystalloid perfusion. Cold crystalloid (lactated Ringer’s solution with mannitol and methylprednisolone or HTK solution in 4°C) is delivered with boluses of 200–400 mL intermittent by another roller pump, The cold crystalloid is administered every 6 min with a flow rate of 300 ml/min for 1–2 min, and the perfusion pressure should be less than 200 mmHg. Ultrafiltration and rewarm can be performed to alleviate the hemodilution and hypothermia due to the massive cold crystalloid solution perfusion. The urine volumes of renal in the periods of renal perfusion and post reimplantation should be evaluated, and diuretics can be administrated.

### Switch to cardiopulmonary bypass from left heart bypass

The switch is implemented in the condition that the pulmonary function and cardiac function are far from covering the need for LHB. Our LHB circuit is integrated with reserved pipelines that can be switched to conventional CPB easily. Stop the LHB circulation, cut line C into two two-port C1 and C2, and C1 connects to venous cannulation or cavoatrial cannulation as venous drainage while port C2 connects to another arterial cannulation as perfusion line after cannulate is completed. move down the hard-shell reservoir (A in Figure 6), which is beneficial to the blood gravity drainage. The site of D can be separated into two ports to connect to the oxygenator integrated with an arterial filter (Terumo, Japan), and empty the gas through the recycle line. Additional heparin is administrated to take the ACT more than 480 s. Run the centrifugal pump, release the tube clump C1 and C4, release the tube clump C3, and the CPB can conduct again.

### The application of the left heart bypass circuit in thoracoabdominal aortic aneurysm repair

We retrospectively analyzed the clinical data of patients undergoing TAAA repair under LHB from 2017 to 2022. This retrospective study was approved by the Committee on the Medical Ethics of Xiangya Hospital, Central South University (No. 202101022), and the requirement for informed consent was waived because of the retrospective design. Trial registration was performed in the Chinese Clinical Trial Registry^1^ (No. ChiCTR2200058222).

We analyzed 26 patients that received TAAA repair under the modular multifunctional LHB from January 2018-March 2022 in Xiangya Hospital, Central South University. The demographic characteristics and extent type of TAAA were shown in Table 1. All patients were managed by the same team of anesthesiologists, surgeons, and perfusionists during the operation. LHB was performed for all patients, while selective visceral perfusion and renal perfusion were applied for the patient Extent II and III TAAA patients. The operative characteristics were shown in Table 2. The average LHB time was206.7 ± 78.60 min, and the operation time was587.8 ± 213.0 min. The renal perfusion performed for Extent II and III patients were 1,810 ± 742 ml with 5.8 ± 1.6 times. The lowest temperature during bypass was 33.0 ± 1.0 °C, which was mainly presented at the initiation of LHB, then the temperature would be enhanced by the heat exchanger, and reached 36.56 ± 0.4 at the termination of LHB. Meanwhile, the lactate concentration (Lac) was 6.9 ± 4.1 mmol/L at the termination of LHB, and 6.9 ± 4.9 mmol/L at the termination of the operation. The volume of ultrafiltration was high to 7,071.1 ± 5,315.0 ml while the urine volume was 1,242.3 ± 1,421.4 ml.

**TABLE 1:** Preoperative patient characteristics.

Variables (n = 26)	n/Medium (IQR)	(%)/Mean±SD
Age (years)	50.0 (38.0-61.0)	49.5±15.1
Male	22	84.62
High (cm)	168.0 (160.5-171.0)	168.3±7.9
weight (kg)	63.5 (55.0-72.0)	63.4±10.0
BMI	22.2 (20.2-25.5)	22.4±3.4
Extent		0
TAAA II	18	69.23
TAAA III	1	3.85
TAAA IV	0	0
TAAA V	7	26.92

**TABLE 2:** Operative characteristics of patients.

Variables (n = 26)	Medium (IQR)	Mean±SD
LHB time (min)	211.5 (159.8-251.0)	206.7±78.6
Operation time (min)	650.0 (472.0-740.0)	587.8±213.0
Renal perfusion times	5.5 (5.0-7.0)	5.8±1.6
Solution volume of renal perfusion(ml)	2000.0 (1462.5-2400.0)	1810.0±742.0
**Temperature (°C)**		
Lowest in LHB	34.0 (33.1-34.5)	33.0±1.0
Termination of LHB	36.7 (36.4-36.8)	36.6±0.4
**HB (g/L)**		
Termination of LHB	83.5 (68.3-93.0)	82.0±18.8
Termination of operation	93.0 (80.5-99.0)	88.0±23.8
**Lac (mmol/L)**		
Termination of LHB	6.2 (3.3-10.8)	6.9±4.1
Termination of operation	5.1 (2.9-10.5)	6.9±4.9
Ultrafiltration volume (ml)	5250.0 (2525.0-11000.0)	7071.1±5315.0
Urine volume (ml)	800.0 (412.5-1200.0)	1242.3±1421.4

HB: Hemoglobin; Lac: Lactate.

The results of blood product transfusion were listed in Table 3. The blood transfusion was performed for all erythrocytes was 12.3 ± 9.7 Units, apheresis platelets were 0.9 ± 1.1 Units, cryoprecipitate was 1.2 ± 1.0 Units, plasma was 965.0 ± 859.2 ml and autologous blood was 1,546.5 ± 1,100.0 ml.

**TABLE 3:** The transfusions of blood products in operation.

Variables	Medium (IQR)	Mean±SD
Erythrocyte (Unit)	9.5 (4.0-19.5)	12.3±9.7
*Apheresisplatelets* (Unit)	1.0 (0.0-1.0)	0.9±1.1
Cryoprecipitate (Unit)	1.0 (1.0-1.0)	1.2±1.0
Plasma (ml)	700.0 (300.0-1500.0)	965.0±859.2
Autologous blood(ml)	1400.0 (1000.0-1625.0)	1546.5±1100.0

Finally, 22 patients were cured and discharged from the hospital, one of the 26 patients died in the operation due to bleeding, and three died in the hospital after the repair. No more patients died during the 30-day postoperative. Two patients required re-exploration for bleeding. The permanent spinal cord deficit appeared in two patients, and one was paraparesis while another one recovered. One patient had a stroke after surgery. Three patients who got acute renal injury (AKI) need hemodialysis. Pneumothorax was the most frequent adverse event after repair with nine patients. The causes of death were postoperative rupture, multiple organ dysfunction syndromes, and severe infection, and all of them were Extent II with a long length of surgery and LHB duration. The postoperative mechanical ventilation time was 20.6 ± 16.1 h, the length of ICU stay was 72.5 ± 46.5 h, and hospital stay time was 13.8 ± 7.1 days for the patients cured and discharged (Table 4). Meanwhile, the laboratory test results including blood routine test, liver and kidney function, electrolyte, myocardial enzyme, and N-terminal pro-B type natriuretic peptide (NT-proBNP) were collected and analyzed with paired *t*-tests for the preoperative and the first day of postoperative data. The results showed that Hb and Platelets were significantly decreased after surgery, and NT-proBNP, Urea, creatinine (Cre), direct bilirubin (DBil), Total bile acid (TBA), lactate dehydrogenase (LHD), creatine kinase (CK), Myoglobin (Mb), prothrombin time (PT), international normalized ratio (INR), activated partial thromboplastin time (APTT), thrombin time (TT), and fibrinogen (FIB), D-Dimer (D2) were rapidly increased. No significant difference was found in the comparison of the levels of Troponin I, Uric acid, alanine aminotransferase (ALT), and aspartate aminotransferase (AST), and creatine kinase-*MB* isoenzyme (*CK*-*MB*). The details were listed in Table 5.

**TABLE 4:** The postoperative outcomes.

Variables (n = 26)	n/Medium (IQR)	(%)/Mean±SD
Mechanical ventilation time (h)	13.0 (8.4-34.8)	20.6 ± 16.1
ICU stay(h)	74.0 (40.9-89.5)	72.47 ± 46.5
Hospital stay (day)	13.0 (10.0-17.0)	13.8 ± 7.10
Intraoperative mortality	1	3.85
In-hospital mortality	4	15.4
30-day in-hospital mortality	4	15.4
Permanent spinal cord deficit	2	7.69
Paraparesis	1	3.85
Stroke	1	3.85
AMI	0	0
AKI necessitating hemodialysis	2	7.69
Re-exploration	1	3.85
MODS	2	7.69
Pneumothorax	9	34.6

AMI: Acute myocardial infarction; AKI: Acute renal injury; MODS: multiple organ dysfunction syndromes.

**TABLE 5:** The preoperative and postoperative laboratory tests outcomes.

Variables		Medium(IQR)	Mean±SD
Hb (g/L)	Preoperative	122.5 (99.8-127.3)	115.0±23.1
	Postoperative	103.0 (84.0-110.5)	96.6±17.6
*Platelets* (10^12^/L)	Preoperative	192.5 (159.8-215.0)	202.0±73.8
	Postoperative	69.0 (54.5-104.5)	94.3±69.1
Troponin I (ng/ml)	Preoperative	0.006 (0.001-0.010)	0.010±0.010
	Postoperative	0.186 (0.065-0.300)	0.250±0.250
NT-proBNP (ng/L)	Preoperative	138.0 (59.7-300.0)	482.0±769.8
	Postoperative	337.0 (187.0-758.2)	602.3±724.4
Urea (mmol/L)	Preoperative	5.47 (4.74-7.36)	6.68±3.73
	Postoperative	8.00 (6.77-11.13)	10.29±5.46
Cre (μmol/L)	Preoperative	94.9 (74.5-117.3)	138.2±195.2
	Postoperative	159.8 (118.1-175.0)	187.7±192.3
Uric acid (μmol/L)	Preoperative	372.1 (278.2-451.6)	371.7±144.1
	Postoperative	359.4 (255.5-411.6)	339.2±117.4
ALT (U/L)	Preoperative	14.05 (10.8-17.5)	16.7±11.9
	Postoperative	28.7 (18.6-47.0)	144.2±373.6
AST (U/L)	Preoperative	17.0 (15.1-22.2)	23.2±18.8
	Postoperative	93.4 (58.6-132.8)	256.9±591.7
DBil (μmol/L)	Preoperative	4.6 (2.5-6.2)	5.7±6.2
	Postoperative	16.2 (10.0-22.3)	20.5±20.2
TBA (μmol/L)	Preoperative	9.6 (5.6-12.8)	12.2±11.8
	Postoperative	32.2 (23.6-63.3)	45.2±35.5
LHD (μmol/L)	Preoperative	186.0 (156.0-210.0)	186.9±45.7
	Postoperative	622.0 (464.2-789.5)	886.6±968.6
CK (μmol/L)	Preoperative	60.1 (51.3-81.2)	84.6±82.5
	Postoperative	2779.0 (1853.7-5011.5)	3577.1±2674.4
CK-MB (μmol/L)	Preoperative	8.9 (7.5-11.9)	12.1±11.8
	Postoperative	56.8 (36.0-82.9)	157.9±453.8
Mb (μmol/L)	Preoperative	26.9 (19.8-35.6)	38.7±50.1
	Postoperative	873.7 (444.3-3145.2)	3603.4±6110.5
PT (S)	Preoperative	13.2 (12.0-13.8)	13.1±1.3
	Postoperative	14.1 (13.3-17.6)	15.4±3.2
INR	Preoperative	1.07 (1.00-1.11)	1.06±0.10
	Postoperative	1.19 (1.09-1.50)	1.32±0.31
APTT (S)	Preoperative	33.4 (30.0-37.5)	33.3±5.2
	Postoperative	38.5 (34.2-55.9)	51.7±29.9
TT (S)	Preoperative	16.3 (15.5-17.1)	16.3±1.4
	Postoperative	20.1 (17.5-28.0)	28.2±19.6
FIB (g/L)	Preoperative	3.6 (2.9-4.7)	4.2±2.0
	Postoperative	2.3 (1.9-2.7)	2.7±1.6
D2 (mg/L)	Preoperative	1.3 (0.5-2.2)	1.7±1.7
	Postoperative	2.2 (1.1-7.2)	6.9±10.3

HB: Hemoglobin; NT-proBNP: N-terminal pro-B type natriuretic peptide;Cre: creatinine, DBil: Direct bilirubin; TBA: Total bile acid; ALT: alanine aminotransferase, AST: aspartate aminotransferase; LHD: lactate dehydrogenase; CK: creatine kinase; Mb: Myoglobin; CK-MB: creatine kinase-MB isoenzyme; PT: Prothrombin time; INR: international normalized ratio; APTT: Activated partial thromboplastin time; TT: Thrombin time; FIB: fibrinogen; D2: D-Dimer.

## Discussion

As a non-high-volume center, we achieved acceptable clinical outcomes for patients who received open TAAA repair under LHB. The in-hospital mortality and 30-day postoperative mortality were 15.4%, and the postoperative incidences of paraparesis (4%), stroke (4%), and AKI need hemodialysis (12%) were not particularly high, based on the limited patient’ sample size in short research period duration. The mortality was 15.4%, which may blame the following factors. The characteristics of patients indicated that high to 69% of patients suffering from type II TAAA, which involves the reconstruction of descending thoracic aorta and abdominal aortic aorta with long-lasting anastomoses and a massive trauma for the patient. Moreover, more patients failed endovascular repair and re-intervention after endovascular repair cases, which made the surgical procedure more complicated. The integrated management of LHB including spinal cord protection and selective visceral perfusion and renal perfusion may contribute to the favorable results of our research.

The morbidity and mortality of the open TAAA repair continue to remain considerable, despite the advances in open surgical techniques. In-hospital mortality ranges between 2.3% (10) and 32.7% (11). Meta-analysis indicated that the total pooled in-hospital mortality rate was 11.26% among all studies including all Crawford types, and was 10.32% for Crawford types II (12). The overall in-hospital mortality of TAAA repair was reported to be more than 19% in the United States (13–15). The high incidence of severe adverse events including spinal cord ischemia, respiratory complications, permanent postoperative renal dialysis, stroke rate, and cardiac events also affect the clinical outcome. Meta-regression evidenced that the mortality had a statistically significant inverse association with the volume of the TAAA center (12). The patients who accepted TAAA repair in high-volume centers have reported excellent results: lower rate of in-hospital mortality and postoperative complications (6, 8, 15). Moreover, the rate of cardiac complications and gastrointestinal complications, as well as blood transfusion rates were also lower for patients operated on at high-volume centers (15). A volume-outcome analysis revealed that high hospital volume is associated with decreased mortality after AAA repair (16). The mortality was significantly inverse correlated with the annual open TAAA repair volume (15). The reported significant predictors of mortality in this study were age older than 65 years (OR 2.78) and emergent presentation (OR 2.41). Other significant predictors of mortality were urgent presentation (OR 1.58), use of extracorporeal circulation (OR 1.38), and annual TAAA volume (15). As a low-volume TAAA center, the morbidity and mortality in our hospital still have potential improvement.

Both open surgical and endovascular repair of TAAA achieved excellent midterm results regarding mortality and the overall survival of patients (17). TAAA has increased markedly, the endovascular repair was increased frequently, while the number of open repairs has declined in the endovascular era (18). The surgeon and hospital volume of open TAAA repairs has decreased considerably over the last two decades, subsequently leading to increased numbers of centers offering endovascular repair (18). However, the endovascular TAAA repair is associated with higher rates of re-intervention (19, 20), and increased early expenses (21). Open repair was performed in limited centers, and always performed in these conditions: complex aortic pathology, unsuitable for endovascular repair, and most reported open repairs were performed in high volume centers with an experienced surgical team (15, 18). It is undisputed that open TAAA repair is a valuable choice for aortic infection, a bailout treatment for complex anatomy, failed endovascular repair and re-intervention after endovascular repair (22). Meanwhile, it is strongly recommended for patients suffering from connective tissue disease according to the current guidelines of the European Society of Vascular Surgery (23).

LHB and CPB with or without hypothermic circulatory arrest, which is a highly invasive procedure may contribute to morbidity and mortality (9). A meta-analysis including three trials indicated that both LHB and CPB with hypothermic circulatory arrest provided adequate organ protection and equivalent clinical outcomes for open TAAA repair (24). CPB with a hypothermic circulatory arrest can afford a clean surgical field for repair (24). However, serious consequences of hypothermic circulatory arrest include coagulopathy, and ischemia/reperfusion-related injury (25, 26). Hypothermic causes coagulopathy, which is due to both CPB and hypothermia, may result in life-threatening bleeding (26, 27). Moreover, hypothermic circulatory arrest increased the risk of low cardiac output syndrome and prolonged ventilator support duration (28).

Overall, LHB is achieved using a temporary bypass from the left atrium to either the distal descending thoracic aorta or the femoral artery with a closed-circuit in-line centrifugal pump (29). LHB can provide distal aortic perfusion while the aorta is cross-clamped, thereby decreasing the ischemic times of distal organs, particularly the spinal cord. Meanwhile, selective visceral perfusion and renal perfusion can enhance the perfusion of gastrointestinal organs and kidneys.

LHB, which requires limited anticoagulation, and mild passive hypothermia, could significantly diminish the influence on physiological functions. LHB appears to provide the greatest benefit to patients undergoing the more extensive repairs and reduced the incidence of spinal cord deficits in patients undergoing extent II repairs (30). The combination of LHB and cerebrospinal fluid drainage may enhance spinal cord protection beyond that provided by either adjunct alone (9, 31). New research found that aorto-iliac bypass could offer sufficient distal aortic perfusion without complex perfusion skills, and was regarded as a substitute for CPB and LHB in spinal cord protection (32). However, the difference between aorto-iliac bypass and LHB on TAAA repair is still unclear due to the limitation of direct comparison data. TAAA repair under aorto-iliac bypass needs systemic heparinisation with heparin 3 mg/kg to acquire the ACT > 480 S, rather than 1.5 mg/kg for LHB. Meanwhile, the maintenance of body temperature and hemodynamic stability is more difficult in aorto-iliac bypass and has a higher technical requirement of the vascular anastomosis to reduce the proximal aortic cross-clamp time within the safe limit (33), because aorto-iliac bypass cannot offer distal aortic perfusion during the period of proximal aortic anastomosis (32).

The traditional LHB circuit for the TAAA repair typically involves a simple circuit consisting of inflow and outflow lines, and a centrifugal head. The blood volume fluctuation is significant, and massive fluid and blood replacement are required rapidly, as a consequence of severe bleeding during extensive exposure and reconstruction of the thoracoabdominal aorta and its aortic branches. So excessive hemodilution, hypotension, and hypothermia may occur, even resulting in overwhelming hemodynamics changes. These conditions are a common phenomenon in the early stage of a low-volume center. Ultrafilter, heater exchanger, arterial filter, and rapid-infusion circuit were added to the classical LHB circuit to diminish to hazard the of profuse bleeding due to operation. The heater exchanger contributes to the temperature regulation; The ultrafilter, arterial filter, and rapid-infusion system facilitated the blood volume management and are useful to maintain hemodynamic stability.

Our modular multifunctional LHB circuit can offer effective distal aortic perfusion during the period of proximal aortic anastomosis, which can afford sufficient time for anastomosis. The integrated rapid infusion system can maintain the systemic blood volume when hemorrhaging to reduce hemodynamic fluctuations. Meanwhile, ultrafiltration is effective to achieve hemoconcentration and hemodilution after hemorrhage and rapid infusion. The collection of shed blood from the surgical field and rapid fusion system. The combination of a rapid infusion system and ultrafiltration can take over the function of an additional cell salvage device, and retain the other components than erythrocytes to a maximum extent. Moreover, the accessibility of modified LHB circuit switching to CPB reduces the incidence of operation errors and time-saving, which may be significantly meaningful in the condition that the cardiopulmonary function can not be competent for LHB. Our circuit needs more consumables including ultrafilter, and arterial filter, which may increase the cost. However, the cost of modified LHB is lower than traditional CPB, which has the essential cost of an *oxygenator*. The cost difference between the new circuit versus the traditional circuit is unclear due to the limited data. The closed-circuit system of LHB cannot respond to sudden hemodynamic fluctuations during aortic clamping or catastrophic bleeding, so the rapid-infuser system and another infusion system should be implemented. Recent research found the reservoir-added centrifugal pump circuit system was an effective perfusion system for blood infusion to reduce hemodynamic fluctuations (34). This research also modified the centrifugal system to facilitate hemodynamic management, and the temperature may be difficult to maintain in the normal range. Our LHB system provides means to achieve rapid infusion, which includes the rapid infuser system and reservoir-added centrifugal pump. Meanwhile, temperature management is easier under the use of a heat exchanger. Moreover, ultrafiltration can be performed in our system to improve blood concentration effectively. The comparison of classical LHB circuit, modified LHB circuit, and classical CPB circuit for TAAA was listed in Supplementary Table 1.

The maximum infusion flow rate of the rapid infuser system is 1,000 ml/min, the actual speed is reduced due to the tube diameter and fusion pressure. The is hard to afford the complete transfusion demand when severe bleeding occurred in actual practice. The volume in the hard-shell reservoir A in Figure 6 can be rapidly infused into the LHB circuit to achieve the blood volume supplement. The shed blood also can be collected to another hard-shell reservoir timely through the sucker lines, and pumped into the rapid infuser system after ultrafiltration by ultrafilter and remove microemboli by an arterial filter. A hemoperfusion cartridge also can be paralleled to the ultrafiltration line in the LHB circuit to reduce the systemic inflammatory responses (35).

As we know and mentioned above, cardiac and pulmonary functions are essential for the performance of LHB. The CPB with hypothermic circulatory arrest is the ultimate option for TAAA repair and should be switched from LHB if the cardiac and pulmonary functions are hard to meet the needs of LHB. The reserved cannulation lines for venous drainage and arterial perfusion, and reserved tube for oxygenator connection, make the switch easily achieved.

Spinal cord ischaemia is another life-threatening event related to increased mortality after complex aortic surgery. Research reported the total incidence of spinal cord ischaemia was 8.6% (12). The incidence of paraparesis own to spinal cord deficit was favorably low in our center. Cerebrospinal fluid drainage and intercostal artery re-implantation are important spinal cord protection strategies (6, 36). The spinal cord perfusion is enhanced when augmented distal aortic perfusion is below the level of proximal aortic cross-clamp, maintaining spinal cord perfusion pressure provides acceptable outcomes. which is another method of protecting the spinal cord against ischemia (10). In our center, cerebrospinal fluid drainage, intercostal artery re-implantation, and enhanced MAP were performed.

The circuit for selective visceral perfusion and kidney perfusion is important for the patients’ need to reconstruct the renal arteries, celiac axis, and the SMA. Selective visceral perfusion and kidney perfusion are essential principles for extent II, III, or IV TAAA patients during the abdominal aorta reconstruction, which aims to minimize the duration of abdominal-organ ischemia and reduce the incidence of renal and hepatic insufficiency, coagulopathy, and bowel ischemia. Postoperative acute kidney injury (AKI) is a common complication and is a significant risk factor for mid-term survival in patients undergoing TAAA repair. Preventing AKI can modify the mid-term survival after TAAA repair (37). Research confirmed that cold crystalloid perfusion during the period of *arterial* occlusion and reconstruction is critical for kidney protection (38). The visceral perfusion is selected to avoid the splanchnic hypoperfusion and to reduce gastrointestinal complications. Gastrointestinal complications are a greater risk factor for mortality and require secondary interventions, longer intensive care unit (ICU) stay, and hospital stays (39). The mid and long-term survival was markedly poorer for patients who developed gastrointestinal complications (36).

The advantages of LHB include augmentation of distal perfusion and reduction of left ventricular strain by decreasing preload and afterload (40). Moreover, the heparin dose is significantly reduced to 1.5 mg/kg with ACT > 250 s under LHB without an oxygenator, which may contribute to less surgical bleeding (40). LHB generally lacks an oxygenator, so maintenance of oxygenation is important during single-lung ventilation. LHB remains controversial when it is used as extracorporeal circulatory support in patients with preoperative pulmonary risks (40–42). Some studies have suggested the use of a CPB or an oxygenator during LHB to achieve better oxygenation in patients with impaired pulmonary function (41, 42). The research found that LHB improved arterial oxygenation during single-lung ventilation in open TAAA repair (40, 43). The marked improvement in oxygenation was due to improvements in ventilation/perfusion mismatch and the pulmonary shunt elicited by the inserting an inflow cannula via the left pulmonary vein (44). However, there are some limitations to our present study. First, no comparative quantitative data to clarify the accessibility, effectiveness, and cost-benefit differences of our modular multifunctional LHB circuit from the traditional LHB circuit. Secondly, our experience is based on retrospectively collected data from a limited number of patients. Thus, more cases are also needed to evaluate the clinical outcomes. Finally, the circuit looks rather complicated, with too many details including cut points and connections. So, a visual guide for the assembly and use of this circuit is provided as the supplementary material (Supplementary Video 1).

In conclusion, the modular LHB circuit system is an effective, practical, and multifunctional perfusion system. The circuit can meet the needs of LHB and quickly switch to conventional CPB. This circuit also made the assembly of the LHB circuit more easily, and more efficient, which may contribute to the TAAA repair operation performed in lower volume centers easily.

## Data availability statement

The original contributions presented in this study are included in the article/Supplementary material, further inquiries can be directed to the corresponding author.

## Ethics statement

The studies involving human participants were reviewed and approved by Committee on the Medical Ethics of Xiangya Hospital, Central South University. Written informed consent for participation was not required for this study in accordance with the national legislation and the institutional requirements.

## Author contributions

CZ and LH: conceptualization. XC, QH, and FL: methodology. XC and CZ: software. CZ, QH, and EW: validation. ZY and CZ: formal analysis, writing—review and editing. QH, EW, and LD: investigation. CZ: resources, data curation, project administration, funding acquisition, and visualization. LH and JH: writing—original draft preparation. LH: supervision. All authors have read and agreed to the published version of the manuscript.

## Footnotes

1
http://www.chictr.org.cn
