# Supplementary material for: The application of modular multifunctional left heart bypass circuit system integrated with ultrafiltration in thoracoabdominal aortic aneurysm repair
Source: Front Cardiovasc Med. 2022 Sep 21;9:944287. doi: 10.3389/fcvm.2022.944287 (PMC9534546; doi:10.3389/fcvm.2022.944287)
Supplement: Supplementary file 1 [file Table_1.docx]

Supplementary table 1: The comparison of classical LHB circuit, modified LHB circuit, and classical CPB circuit for TAAA

| Perfusion circuit | Ultrafilter | Sucker for the shed blood | Rapid infusion | Switch to CPB | ACT(S) | Oxygenator | Heat exchanger extra-oxygenator | Sites of cannulation for drainage | Sites of cannulation for perfusion | Temperature |  |
| --- | --- | --- | --- | --- | --- | --- | --- | --- | --- | --- | --- |
| Standard LHB | No | No | No | Reassemble the CPB circuit | >250 | No | Yes | Left pulmonary vein | The femoral artery, iliac artery, abdominal aorta | Normal temperature | |
| Modified LHB circuit | Yes | Yes | Yes | Quickly switch to CPB using reserved pipelines for cannulation and oxygenator connection | >250 | No | Yes | Left pulmonary vein | The femoral artery, iliac artery, abdominal aorta | Normal temperature | |
| CPB with/out HCA | Yes | Yes | Yes | No | >480 | Yes | No | Superior inferior vena cava, femoral vein or cavoatrial cannulation | The femoral artery, iliac artery, abdominal aorta | Mild hypothermia or DHCA | |
